# Supplementary material for: Multi‐dataset identification of innovative feature genes and molecular mechanisms in keratoconus
Source: J Cell Mol Med. 2024 Sep 19;28(18):e70079. doi: 10.1111/jcmm.70079 (PMC11412914; doi:10.1111/jcmm.70079)
Supplement: Supplementary file 2 — Material S2. [file JCMM-28-e70079-s002.zip › 7.GSEA analysis/6.SNORA74B/KEGG_ENDOCYTOSIS.html]

Details for gene set KEGG\_ENDOCYTOSIS[GSEA]

|  || Dataset | SNORA74B.group.cls#h\_versus\_l |
| Phenotype | group.cls#h\_versus\_l |
| Upregulated in class | l |
| GeneSet | KEGG\_ENDOCYTOSIS |
| Enrichment Score (ES) | -0.27082986 |
| Normalized Enrichment Score (NES) | -1.4411823 |
| Nominal p-value | 0.062992126 |
| FDR q-value | 0.32632452 |
| FWER p-Value | 0.861 |
Table: GSEA Results Summary

  

Fig 1: Enrichment plot: KEGG\_ENDOCYTOSIS      
 Profile of the Running ES Score & Positions of GeneSet Members on the Rank Ordered List

  

| SYMBOL | TITLE | RANK IN GENE LIST | RANK METRIC SCORE | RUNNING ES | CORE ENRICHMENT || 1 | CLTC | na | 2065 | 0.366 | -0.0406 | No |
| 2 | RET | na | 3274 | 0.288 | -0.0604 | No |
| 3 | ARAP2 | na | 4059 | 0.258 | -0.0696 | No |
| 4 | PDGFRA | na | 4146 | 0.255 | -0.0587 | No |
| 5 | RAB11FIP4 | na | 4625 | 0.238 | -0.0601 | No |
| 6 | CXCR2 | na | 5077 | 0.223 | -0.0614 | No |
| 7 | CBLB | na | 5386 | 0.212 | -0.0592 | No |
| 8 | ARAP1 | na | 5522 | 0.208 | -0.0522 | No |
| 9 | AGAP2 | na | 5530 | 0.207 | -0.0415 | No |
| 10 | PSD2 | na | 5590 | 0.205 | -0.0325 | No |
| 11 | EPS15 | na | 5797 | 0.199 | -0.0280 | No |
| 12 | ASAP3 | na | 6075 | 0.190 | -0.0260 | No |
| 13 | IGF1R | na | 6399 | 0.180 | -0.0259 | No |
| 14 | PDCD6IP | na | 6563 | 0.175 | -0.0215 | No |
| 15 | IL2RB | na | 6899 | 0.165 | -0.0225 | No |
| 16 | SMAP1 | na | 8324 | 0.129 | -0.0570 | No |
| 17 | HSPA2 | na | 8508 | 0.124 | -0.0558 | No |
| 18 | NEDD4L | na | 8589 | 0.122 | -0.0517 | No |
| 19 | HSPA8 | na | 8644 | 0.121 | -0.0468 | No |
| 20 | CHMP4B | na | 8785 | 0.118 | -0.0447 | No |
| 21 | FGFR4 | na | 9201 | 0.109 | -0.0510 | No |
| 22 | ARRB1 | na | 9230 | 0.109 | -0.0461 | No |
| 23 | EPN3 | na | 10219 | 0.088 | -0.0701 | No |
| 24 | NTRK1 | na | 10524 | 0.083 | -0.0745 | No |
| 25 | AP2A2 | na | 10586 | 0.082 | -0.0720 | No |
| 26 | PIKFYVE | na | 10605 | 0.081 | -0.0682 | No |
| 27 | ARFGAP1 | na | 10846 | 0.077 | -0.0711 | No |
| 28 | ARAP3 | na | 10991 | 0.075 | -0.0713 | No |
| 29 | FGFR3 | na | 11176 | 0.072 | -0.0729 | No |
| 30 | CBL | na | 11198 | 0.072 | -0.0697 | No |
| 31 | PSD4 | na | 11200 | 0.071 | -0.0660 | No |
| 32 | MDM2 | na | 11211 | 0.071 | -0.0626 | No |
| 33 | STAMBP | na | 11233 | 0.071 | -0.0595 | No |
| 34 | PIP4K2B | na | 11358 | 0.069 | -0.0594 | No |
| 35 | RAB11FIP1 | na | 11567 | 0.066 | -0.0620 | No |
| 36 | RAB5C | na | 11668 | 0.065 | -0.0615 | No |
| 37 | VPS37B | na | 11673 | 0.065 | -0.0582 | No |
| 38 | ACAP3 | na | 12032 | 0.059 | -0.0655 | No |
| 39 | PRKCZ | na | 12067 | 0.059 | -0.0633 | No |
| 40 | F2R | na | 12299 | 0.056 | -0.0671 | No |
| 41 | RAB22A | na | 12358 | 0.055 | -0.0659 | No |
| 42 | VPS36 | na | 12573 | 0.052 | -0.0693 | No |
| 43 | DNM2 | na | 12643 | 0.051 | -0.0686 | No |
| 44 | ACAP2 | na | 12759 | 0.050 | -0.0693 | No |
| 45 | HLA-F | na | 13085 | 0.046 | -0.0763 | No |
| 46 | DNM3 | na | 13211 | 0.045 | -0.0776 | No |
| 47 | HLA-B | na | 13306 | 0.044 | -0.0780 | No |
| 48 | ADRB2 | na | 13437 | 0.042 | -0.0796 | No |
| 49 | PSD | na | 13465 | 0.042 | -0.0782 | No |
| 50 | PARD6G | na | 13697 | 0.039 | -0.0828 | No |
| 51 | CBLC | na | 13962 | 0.036 | -0.0885 | No |
| 52 | EPN2 | na | 14069 | 0.035 | -0.0898 | No |
| 53 | EPN1 | na | 14160 | 0.034 | -0.0906 | No |
| 54 | DNM1L | na | 14208 | 0.034 | -0.0902 | No |
| 55 | VPS28 | na | 14451 | 0.031 | -0.0956 | No |
| 56 | CHMP3 | na | 14492 | 0.030 | -0.0952 | No |
| 57 | SH3GLB2 | na | 14724 | 0.027 | -0.1004 | No |
| 58 | GIT1 | na | 14994 | 0.024 | -0.1069 | No |
| 59 | ERBB3 | na | 15019 | 0.024 | -0.1063 | No |
| 60 | PARD3 | na | 15192 | 0.023 | -0.1101 | No |
| 61 | LDLR | na | 15209 | 0.022 | -0.1094 | No |
| 62 | VPS4A | na | 15389 | 0.021 | -0.1135 | No |
| 63 | FLT1 | na | 15419 | 0.020 | -0.1133 | No |
| 64 | IQSEC2 | na | 15490 | 0.020 | -0.1143 | No |
| 65 | ACAP1 | na | 15518 | 0.019 | -0.1140 | No |
| 66 | HLA-E | na | 15707 | 0.018 | -0.1186 | No |
| 67 | ARRB2 | na | 15989 | 0.015 | -0.1259 | No |
| 68 | SNF8 | na | 16138 | 0.013 | -0.1295 | No |
| 69 | CLTA | na | 16174 | 0.013 | -0.1298 | No |
| 70 | TFRC | na | 16223 | 0.012 | -0.1306 | No |
| 71 | VPS25 | na | 16388 | 0.011 | -0.1348 | No |
| 72 | HSPA1A | na | 16403 | 0.010 | -0.1346 | No |
| 73 | CHMP6 | na | 16477 | 0.010 | -0.1362 | No |
| 74 | RAB31 | na | 16588 | 0.009 | -0.1390 | No |
| 75 | EEA1 | na | 16608 | 0.008 | -0.1391 | No |
| 76 | PLD2 | na | 16641 | 0.008 | -0.1396 | No |
| 77 | AP2M1 | na | 16749 | 0.007 | -0.1423 | No |
| 78 | PRKCI | na | 16852 | 0.006 | -0.1450 | No |
| 79 | ARFGAP2 | na | 17019 | 0.005 | -0.1495 | No |
| 80 | STAM2 | na | 17033 | 0.004 | -0.1497 | No |
| 81 | VPS37C | na | 17085 | 0.004 | -0.1510 | No |
| 82 | HGS | na | 17106 | 0.004 | -0.1513 | No |
| 83 | VPS37A | na | 17109 | 0.004 | -0.1512 | No |
| 84 | HSPA1B | na | 17184 | 0.003 | -0.1532 | No |
| 85 | LDLRAP1 | na | 17190 | 0.003 | -0.1532 | No |
| 86 | IQSEC1 | na | 17348 | 0.001 | -0.1577 | No |
| 87 | EGF | na | 17351 | 0.001 | -0.1577 | No |
| 88 | HLA-C | na | 17481 | -0.000 | -0.1614 | No |
| 89 | CHMP2B | na | 17911 | -0.005 | -0.1736 | No |
| 90 | WWP1 | na | 18053 | -0.007 | -0.1773 | No |
| 91 | CHMP4A | na | 18188 | -0.008 | -0.1807 | No |
| 92 | AGAP1 | na | 18211 | -0.008 | -0.1809 | No |
| 93 | SH3GL1 | na | 18302 | -0.009 | -0.1830 | No |
| 94 | HLA-A | na | 18401 | -0.011 | -0.1853 | No |
| 95 | PIP5K1C | na | 18480 | -0.012 | -0.1870 | No |
| 96 | PLD1 | na | 18510 | -0.012 | -0.1872 | No |
| 97 | STAM | na | 18513 | -0.012 | -0.1866 | No |
| 98 | ITCH | na | 18518 | -0.012 | -0.1861 | No |
| 99 | RAB4A | na | 18717 | -0.014 | -0.1911 | No |
| 100 | CDC42 | na | 18770 | -0.015 | -0.1918 | No |
| 101 | AP2S1 | na | 18845 | -0.015 | -0.1932 | No |
| 102 | HLA-G | na | 18916 | -0.016 | -0.1944 | No |
| 103 | ADRB1 | na | 19094 | -0.018 | -0.1985 | No |
| 104 | VPS4B | na | 19153 | -0.019 | -0.1992 | No |
| 105 | CHMP2A | na | 19331 | -0.021 | -0.2032 | No |
| 106 | SH3GLB1 | na | 19436 | -0.022 | -0.2051 | No |
| 107 | EGFR | na | 19596 | -0.024 | -0.2084 | No |
| 108 | HRAS | na | 19699 | -0.025 | -0.2100 | No |
| 109 | GRK6 | na | 19851 | -0.027 | -0.2130 | No |
| 110 | RAB11B | na | 19997 | -0.028 | -0.2157 | No |
| 111 | RUFY1 | na | 20019 | -0.029 | -0.2148 | No |
| 112 | TSG101 | na | 20133 | -0.030 | -0.2165 | No |
| 113 | USP8 | na | 20374 | -0.032 | -0.2218 | No |
| 114 | SH3GL3 | na | 20552 | -0.035 | -0.2251 | No |
| 115 | EHD4 | na | 20704 | -0.036 | -0.2275 | No |
| 116 | RBSN | na | 20955 | -0.039 | -0.2327 | No |
| 117 | GRK5 | na | 21299 | -0.044 | -0.2404 | No |
| 118 | MVB12A | na | 21316 | -0.044 | -0.2385 | No |
| 119 | RAB5A | na | 21434 | -0.045 | -0.2395 | No |
| 120 | VPS37D | na | 21459 | -0.045 | -0.2379 | No |
| 121 | NEDD4 | na | 21641 | -0.047 | -0.2406 | No |
| 122 | EHD1 | na | 21814 | -0.050 | -0.2430 | No |
| 123 | RAB11FIP2 | na | 21927 | -0.051 | -0.2436 | No |
| 124 | VTA1 | na | 22109 | -0.053 | -0.2460 | No |
| 125 | AP2B1 | na | 22298 | -0.055 | -0.2486 | No |
| 126 | CHMP5 | na | 22526 | -0.059 | -0.2520 | No |
| 127 | ARF6 | na | 22549 | -0.059 | -0.2496 | No |
| 128 | TRAF6 | na | 22560 | -0.059 | -0.2468 | No |
| 129 | RAB11A | na | 22712 | -0.061 | -0.2480 | No |
| 130 | ARFGAP3 | na | 23290 | -0.070 | -0.2610 | No |
| 131 | KIT | na | 23499 | -0.073 | -0.2632 | No |
| 132 | CCR5 | na | 23558 | -0.074 | -0.2610 | No |
| 133 | CLTB | na | 23698 | -0.076 | -0.2610 | No |
| 134 | CHMP1B | na | 23948 | -0.080 | -0.2640 | Yes |
| 135 | HSPA1L | na | 24075 | -0.082 | -0.2634 | Yes |
| 136 | DNM1 | na | 24199 | -0.084 | -0.2626 | Yes |
| 137 | FGFR2 | na | 24244 | -0.085 | -0.2594 | Yes |
| 138 | IL2RA | na | 24251 | -0.085 | -0.2551 | Yes |
| 139 | CHMP4C | na | 24325 | -0.086 | -0.2527 | Yes |
| 140 | GIT2 | na | 24471 | -0.089 | -0.2523 | Yes |
| 141 | MVB12B | na | 24698 | -0.093 | -0.2539 | Yes |
| 142 | RAB11FIP3 | na | 24711 | -0.093 | -0.2494 | Yes |
| 143 | SMURF2 | na | 24938 | -0.097 | -0.2508 | Yes |
| 144 | DNAJC6 | na | 24968 | -0.098 | -0.2465 | Yes |
| 145 | RAB5B | na | 25522 | -0.108 | -0.2569 | Yes |
| 146 | PSD3 | na | 25597 | -0.109 | -0.2533 | Yes |
| 147 | SMURF1 | na | 25836 | -0.113 | -0.2542 | Yes |
| 148 | IL2RG | na | 26016 | -0.117 | -0.2533 | Yes |
| 149 | RAB11FIP5 | na | 26039 | -0.118 | -0.2477 | Yes |
| 150 | VPS45 | na | 26189 | -0.121 | -0.2457 | Yes |
| 151 | AP2A1 | na | 26356 | -0.124 | -0.2440 | Yes |
| 152 | EHD2 | na | 26582 | -0.130 | -0.2437 | Yes |
| 153 | RABEP1 | na | 26617 | -0.130 | -0.2379 | Yes |
| 154 | ASAP2 | na | 26620 | -0.131 | -0.2311 | Yes |
| 155 | PARD6A | na | 26746 | -0.134 | -0.2277 | Yes |
| 156 | SMAP2 | na | 27962 | -0.164 | -0.2542 | Yes |
| 157 | IQSEC3 | na | 28254 | -0.172 | -0.2536 | Yes |
| 158 | CSF1R | na | 28378 | -0.176 | -0.2480 | Yes |
| 159 | DAB2 | na | 28411 | -0.177 | -0.2396 | Yes |
| 160 | MET | na | 28809 | -0.189 | -0.2412 | Yes |
| 161 | ERBB4 | na | 29719 | -0.216 | -0.2562 | Yes |
| 162 | SRC | na | 30227 | -0.234 | -0.2585 | Yes |
| 163 | RNF41 | na | 30352 | -0.239 | -0.2496 | Yes |
| 164 | GRK4 | na | 30718 | -0.254 | -0.2468 | Yes |
| 165 | CLTCL1 | na | 30955 | -0.265 | -0.2397 | Yes |
| 166 | SH3GL2 | na | 31096 | -0.273 | -0.2295 | Yes |
| 167 | HSPA6 | na | 31406 | -0.289 | -0.2233 | Yes |
| 168 | EHD3 | na | 31519 | -0.296 | -0.2110 | Yes |
| 169 | KDR | na | 32219 | -0.336 | -0.2136 | Yes |
| 170 | PARD6B | na | 32690 | -0.367 | -0.2080 | Yes |
| 171 | GRK7 | na | 32883 | -0.384 | -0.1934 | Yes |
| 172 | PIP5K1A | na | 33089 | -0.403 | -0.1782 | Yes |
| 173 | GRK1 | na | 33143 | -0.408 | -0.1583 | Yes |
| 174 | ADRB3 | na | 33378 | -0.434 | -0.1423 | Yes |
| 175 | SH3KBP1 | na | 33455 | -0.444 | -0.1212 | Yes |
| 176 | CXCR4 | na | 33720 | -0.481 | -0.1036 | Yes |
| 177 | ASAP1 | na | 33924 | -0.518 | -0.0823 | Yes |
| 178 | CXCR1 | na | 34414 | -0.656 | -0.0621 | Yes |
| 179 | PIP5K1B | na | 34724 | -1.356 | 0.0001 | Yes |
Table: GSEA details [plain text format]

  

Fig 2: KEGG\_ENDOCYTOSIS      
 Blue-Pink O' Gram in the Space of the Analyzed GeneSet

  

Fig 3: KEGG\_ENDOCYTOSIS: Random ES distribution      
 Gene set null distribution of ES for **KEGG\_ENDOCYTOSIS**
